# Factors associated with intrinsic capacity impairment in hospitalized older adults: a latent class analysis

**DOI:** 10.1186/s12877-024-05093-z

**Published:** 2024-06-05

**Authors:** Lingzhi Zhu, Xiaoxing Shen, Xiaolan Shi, Xiaojun Ouyang

**Affiliations:** 1 School of Nursing, Nanjing Medical University, Nanjing, China; 2 Department of Geriatrics, Geriatric Hospital of Nanjing Medical University, Nanjing, China

**Keywords:** Intrinsic capacity, Latent class analysis, Lifestyle, Older adults

## Abstract

**Background:**

Intrinsic capacity (IC) is proposed by the World Health Organization (WHO) to promote healthy aging. Although some studies have examined the factors influencing IC, few studies have comprehensively confirmed lifestyle factors on IC, especially IC impairment patterns. The present study aimed to identify the patterns of IC impairment and explore the lifestyle and other factors associated with different patterns of IC impairment.

**Methods:**

This cross-sectional study was conducted in a Chinese geriatric hospital. IC was evaluated in five domains according to the recommendations of WHO: cognition, locomotion, vitality, sensory and psychological domains. The sociodemographic and health-related characteristics of participants were assessed.The health promoting lifestyle was evaluated using the Health-Promoting Lifestyle Profile-II scale, including nutrition, health responsibility, interpersonal relationships, physical activity, spiritual growth and stress management. We applied latent class analysis to identify IC impairment patterns and compared basic activities of daily living, instrumental activities of daily living, frailty, quality of life and falls among different IC impairment patterns. Multinomial logistic regression analysis was conducted to identify factors influencing the IC impairment patterns.

**Results:**

Among 237 participants included, the latent class analysis identified three patterns of IC impairment: 44.7% high IC (Class 1), 31.2% intermediate IC mainly locomotor impairment (Class 2) and 24.1% low IC mainly cognitive impairment (Class 3). Older adults in class 1 had the best function ability and quality of life, while class 3 had the highest levels of disability and frailty, the poorest quality of life and a higher prevalence of falls. Compared with class 1, older adults with advanced age (*OR* = 22.046, *95%CI*:1.735-280.149), osteoporosis (*OR* = 3.377, *95%CI*:1.161–9.825), and lower scores in physical activity (*OR* = 0.842, *95%CI*:0.749–0.945), stress management (*OR* = 0.762, *95%CI*:0.585–0.993) and social support (*OR* = 0.897, *95%CI*:0.833–0.965) were more likely to belong to the class 2. Simultaneously, compared with class 1, older adults with advanced age (*OR* = 104.435, *95%CI*:6.038-1806.410), stroke (*OR* = 3.877, *95%CI*:1.172–12.823) and lower scores in physical activity (*OR* = 0.784, *95%CI*:0.667–0.922) and social support (*OR* = 0.909, *95%CI*:0.828–0.998) were more likely to be class 3. In addition, compared with class 2, older adults with a lower score in nutrition (*OR* = 0.764, *95%CI*:0.615–0.950) were more likely to belong to the class 3.

**Conclusions:**

This study provides evidence that there are heterogeneous IC impairment patterns in older adults and identifies various associated factors in each pattern, including age, stroke, osteoporosis, social support and lifestyle behaviors such as nutrition, physical activity and stress management. It informs stakeholders on which modifiable factors should be targeted through public health policy or early intervention to promote IC and healthy aging in older adults.

**Supplementary Information:**

The online version contains supplementary material available at 10.1186/s12877-024-05093-z.

## Background

The world’s population is rapidly aging. According to data from World Population Prospects 2022, the proportion of people aged 65 or older reached 9.7% in 2022, increasing to 16.4% in 2050 [1]. Aging related physiological and pathological changes increase the risk of multimorbidity, falls and disability, which challenges the healthcare system and society [2]. As a response to population aging and reducing care dependency, the World Health Organization (WHO) advocates transitioning from a disease-centered model to a model centered on positive functioning for healthy aging, emphasizing the maintenance of functional ability and prevention of capacity loss. Intrinsic capacity (IC) is the composite of an individual’s physical and mental capacities, including vitality, locomotion, cognition, psychology and sensory domains, which interact with environmental factors determining the functional ability of that person [3].

The multidimensional structure of IC has been validated [4, 5], which can predict many adverse outcomes such as falls, quality of life decline, frailty, disability, and death in older adults [6]. However, the mechanism leading to IC impairment is unclear due to its complexity and the result of numerous factors. Si et al. identified that people with favourable early-life factors were more likely to have higher intrinsic capacity in later life [7]. Three cross-sectional studies of large populations have shown that sociodemographic factors (e.g., age, sex, work status, marital state, education level, region, place of residence, and income), chronic diseases (chronic obstructive pulmonary disease, osteoarthritis and chronic neurologic illness) and lifestyles (physical activity, smoking) may cause IC decline [8–10]. Lifestyle intervention, including nutrition, physical activity, well-being, stress management, and sleep, has the potential to prevent functional decline, frailty, and sarcopenia to optimize the trajectory of aging [11]. WHO also reported that 53% of deaths were due to adverse lifestyle behaviors [12]. Individuals who maintain a healthy lifestyle, such as refraining from smoking and heavy drinking, engaging in physical activity and daily intake of fruits and vegetables, have been observed to be associated with better health outcomes, including healthy aging [13], longer life expectancy [14], increase in life-years lived in good health [15] and cognitive health [16]. Furthermore, these associations exist even among individuals 75 years and older, highlighting the need to advocate for favourable lifestyle behaviors even among the oldest population to enhance life expectancy and physical function [17, 18]. Although some studies have investigated the association between a single element of lifestyle, such as physical activity and smoking, with IC, the Health-Promoting Lifestyle Profile II (HPLP-II) provides multi-dimensional assessments of the lifestyle that promotes health. The health promoting lifestyle is a multidimensional pattern of self-organized actions and perceptions that contribute to maintaining or improving individual health, self-realization, and happiness. It includes six dimensions: self-actualization, health responsibility, exercise, nutrition, interpersonal support and stress management [19]. Few studies have investigated the association between the health promoting lifestyle and IC [9, 20]. However, lifestyles are modifiable factors, and adopting strategies focused on promoting a healthy lifestyle could be the most cost-effective way to maintain function among older adults, particularly in countries with limited resources.

Although previous studies investigated factors influencing the number or degree of impaired IC domains [8–10], the IC measurements in these studies were inconsistent with the WHO recommendations. Most importantly, there is a lack of standard operation of the IC score [21]. However, latent class analysis (LCA) may be a good way to solve this problem, which looks at IC impairment from the perspective of “clusters” rather than “numbers”. It can identify different patterns of IC impairment in older adults and ensure IC integrity. LCA, an unsupervised technique, is based on peoples’ different scoring patterns across variables, which could identify subgroups of individuals with shared characteristics [22].

Therefore, the aim of this study is to identify the patterns of IC impairment and explore the lifestyle and other factors associated with different patterns of IC impairment in older hospitalized patients.

## Methods

### Study design and participants

From December 2022 to August 2023, a cross-sectional study was conducted at the Geriatric Hospital of Nanjing Medical University in China. Medical inpatients admitted to the Department of Geriatrics aged ≥ 60 years and willing to participate in our study. The exclusion criteria were as follows: (1) acute conditions (e.g., acute coronary syndrome, acute heart failure, acute cerebrovascular disease, acute exacerbation of chronic obstructive pulmonary disease); (2)inability to complete assessment due to deafness, blindness and severe cognitive impairment diagnosed by a neurologist. Prior to the study, all participants provided the study protocol and granted informed consent. Participants underwent assessments in a stable period of the disease. The questionnaires were assessed by a well-trained nurse who qualified in comprehensive geriatric assessment (CGA) using a face-to-face interview technique. For patients with severe hearing impairment, white boards were used to communicate with them. The objective information of 6 participants with severe hearing impairments was collected from their caregivers. Finally, 10 participants who did not complete IC or Health-promoting lifestyle profile-II assessment were excluded, and 237 participants were recruited, which is more than 10 times the number of variables and meets the requirement for regression analysis [23]. This study was approved by the Ethics Committee of the Geriatric Hospital of Nanjing Medical University (Ethic number: 2022,042), which was performed in accordance with the guidelines outlined in the Declaration of Helsinki.

### Intrinsic capacity

The assessment of IC was based on the WHO Integrated Care for Older People (ICOPE) guideline [24]. IC included cognitive impairment (the Chinese version of the Mini-Mental State Examination score ≤ 17 for illiterate individuals, ≤ 20 for people with primary school education, and ≤ 24 for people with middle school or higher education) [25], vitality impairment (the Short-Form Mini Nutritional Assessment score ≤ 11) [26], locomotion impairment (the Short Physical Performance Battery test score ≤ 9) [27], psychological impairment (the 15-item Geriatric Depression Scale score ≥ 8) [28], and sensory impairment (answer of “yes” to the question “Do you have experienced vision or hearing decline affecting daily life?”) [29]. An impairment in any of the five domains of IC was considered as IC impairment [8].

### Health promoting lifestyle

All participants completed the Chinese version of the Health-Promoting Lifestyle Profile-II (HPLP-IIR) [30], revised by Cao et al., and it was a widely used instrument to evaluate the health behaviors and lifestyles of older adults. HPLP-IIR is a 40-item questionnaire comprising six dimensions: nutrition, health responsibility, interpersonal relationships, physical activity, spiritual growth and stress management. Each item is measured using a four-point Likert scale (1 = never, 2 = sometimes, 3 = often, and 4 = routinely). Higher scores indicate greater adherence to healthy life behaviors.

### Frailty, disability, quality of life and falls

The FRAIL scale was used to assess frailty based on five criteria: fatigue (Do you feel tired most of the time?), resistance (Do you have difficulty climbing 10 steps of stairs on your own and without using aids?), ambulation (Do you have difficulty walking 500 m on your own and without using aids?), illness (Do you have more than 5 of the following diseases: hypertension, diabetes, cancer, chronic lung disease, heart attack, congestive heart failure, angina, asthma, arthritis, stroke, and kidney disease?), and loss of weight (Do you lose weight > 5% over a past year?). Each criterion answered “yes” was given a score of “1”, otherwise “0”. Frail scale scores ranged from 0 to 5, with higher scores indicating higher frailty [31]. Disability was assessed using basic activities of daily living (BADL) and instrumental activities of daily living (IADL). BADL was assessed using the Barthel Index and ranges from 0 to 100, which includes grooming, bathing, toileting, bowel control, bladder control, feeding, dressing, stair climbing, chair transfer and ambulation [32]. Higher scores indicate more independence. IADL ranges from 0 to 8, including the ability to use a phone, shopping, meal preparation, housekeeping, laundry, the model of transportation, taking prescribed medications, and the ability to handle finances [33]. Higher scores indicate better instrumental living performance. To assess the quality of life (QOL), we used the three-level EuroQol-5D scale (EQ-5D-3 L), including two components: a descriptive system and a visual analogue scale (VAS) [34]. The descriptive system includes five dimensions (mobility, self-care, usual activities, pain/discomfort, and anxiety/depression) with three problem levels (none, moderate, or extreme) and the EQ-5D index was derived based on the Chinese set of EQ-5D-3 L values ranging from − 0.149 to 1 [35]. A higher score indicates better QOL. Self-reported health status was recorded according to the score of the visual analogue scale (EQ-VAS), ranging from 0 (“the worst health you can imagine”) to 100 (“the best health you can imagine”). The falls were evaluated using the following question: “Have you fallen in the last 1 year ?”

### Sociodemographic and health-related characteristics

Sociodemographic characteristics included age (60–74 years, 75–89 years, ≥ 90years), gender, educational level (< 9 years, 9–12 years or > 12 years), monthly personal income (< 5000 ¥, ≥ 5000 ¥), living alone (yes or no) and social support. The Social Support Rate Scale (SSRS) was used to assess social support, including 10 items and three dimensions: objective social support, subjective social support, and the utilization of social support, with higher scores indicating better social support [36]. Health-related characteristics included current smoking (yes or no), sleeping hours, polypharmacy (yes or no), Charlson Comorbidity Index (CCI), and chronic diseases. Regular intake of five or more medications was considered polypharmacy [37]. Polypharmacy, CCI and chronic diseases (history of stroke/hypertension/diabetes/coronary heart disease/chronic obstructive pulmonary disease (COPD)/osteoporosis/cancer) were obtained from medical records.

### Statistical analysis

Statistical data processing was performed using Mplus (version 8) and SPSS (version 25). Latent class analysis was performed with Mplus to identify the latent classes of IC impairment. We reported model fit indices for each model, such as the value of the Akaike Information Criterion (AIC), Bayesian Information Criterion (BIC), Sample-size Adjusted BIC (SABIC), Entropy, and Lo-Mendell-Rubin (LMR) [38]. Lower AIC, BIC and SABIC values and higher entropy indicate better model fit. The entropy value above 0.8 is considered acceptable, indicating the accuracy of classification is greater than 90%. If the P values of LMR < 0.05, it means the k class model is better than the k-1 class model. In addition to model fit indices, the simplicity and interpretability of the model as well as clinical insights should also be taken into consideration.

Descriptive statistics and further analysis were performed with SPSS. Continuous variables with normal distribution were expressed as means ± standard deviations, and non-normal distribution was expressed as median and interquartile range. Categorical variables were reported as frequencies and percentages. After the appropriate number of latent classes was determined, the characteristics between different classes were compared using the Kruskal-Wallis test, one-way analysis of variance, or the chi-square test. Subsequently, variables with a *P*-value < 0.05 in univariate analysis were selected for multivariate multinomial logistic regression to explore the factors influencing different classes. A *P*‐value of < 0.05 was considered statistically significant.

## Results

### Sample characteristics

A total of 237 participants (mean age 80.59 ± 8.85 years[range, 60-101years]) were included in this study and 63.3% were male. The prevalence of IC impairment was 69.6% and impairment in cognition, locomotion, psychological, vitality, and sensory domains was 27%, 52.7%, 3%, 25.3% and 39.7%, respectively. The characteristics of participants are presented in Table 1.

**Table 1:** Baseline characteristics of the sample (*N* = 237)

Variables	Mean ± SD or M (P25, P75)	*N* (%)
Age (years)		
60–74		61 (25.7%)
75–89		132 (55.7%)
≥ 90		44 (18.6%)
Gender		
Male		150 (63.3%)
Female		87 (36.7%)
Education level (years)		
< 9		53 (22.4%)
9–12		67 (28.3%)
> 12		117 (49.4%)
Monthly personal income		
< 5000 ¥ (approximately 700 US$)		50 (21.1%)
≥ 5000 ¥ (approximately 700 US$)		187 (78.9%)
Living alone		
No		204 (86.1%)
Yes		33 (13.9%)
Current smoking		
No		212 (89.5%)
Yes		25 (10.5%)
Sleeping hours	6 (5, 7)	
Health-promoting lifestyle profile-II		
Nutrition	21 (19, 22)	
Health responsibility	21 (18, 25)	
Interpersonal relationships	13 (10, 16)	
Physical activity	14 (10, 18)	
Spiritual growth	9 (6.5, 11)	
Stress management	14 (13,14)	
Polypharmacy		
No		84 (35.4%)
Yes		153 (64.6%)
CCI	1 (0, 2)	
Stroke		
No		150 (63.3%)
Yes		87 (36.7%)
Hypertension		
No		50 (21.1%)
Yes		187 (78.9%)
Diabetes		
No		146 (61.6%)
Yes		91 (38.4%)
Coronary heart disease		
No		157 (66.2%)
Yes		80 (33.8%)
COPD		
No		226 (95.4%)
Yes		11 (4.6%)
Osteoporosis		
No		185 (78.1%)
Yes		52 (21.9%)
Cancer		
No		222 (93.7%)
Yes		15 (6.3%)
SSRS	36.23 ± 7.421	
Impaired IC domains		
Cognitive impairment		64 (27%)
Locomotion impairment		125 (52.7%)
Psychological impairment		7 (3.0%)
Vitality impairment		60 (25.3%)
Sensory impairment		94 (39.7%)
Number of impaired IC domains		
0		72 (30.4%)
1		58 (24.5%)
2		51 (21.5%)
3		36 (15.2%)
4		18 (7.6%)
5		2 (0.8%)

CCI Charlson Comorbidity Index, COPD chronic obstructive pulmonary disease, SSRS Social support rate scale, IC intrinsic capacity, M median, SD standard deviation

### Latent profiles of IC impairment

The fit indices from the 1- to 4-class models are presented in Table 2. The 2-class model performed better on model fit indices, because it had the lowest AIC/BIC/aBIC values and had a significant of LMR results. Although the 3-class model was not statistically better than the 2-class model, it had the highest entropy value and was more clinically meaningful and interpretable. Finally, the 3-class model was selected to define IC impairment patterns in this study.

**Table 2:** Fit indices of latent class analysis of the IC impairment

Model	AIC	BIC	SABIC	Entropy	LMR(*P*)	Classification probability
1	1263.954	1281.294	1265.446			1
2	1177.443	1215.592	1180.726	0.621	< 0.001	0.43882/ 0.56118
3	1184.945	1243.902	1190.018	0.835	0.3925	0.31224/0.24051/0.44726
4	1192.634	1272.400	1199.498	0.705	0.1041	0.01266/0.49789/0.40506/0.08439

AIC Akaike information criterion, BIC Bayesian information criterion, SABIC Sample-size Adjusted BIC, LMR Lo-Mendell-Rubin

**Fig. 1:**
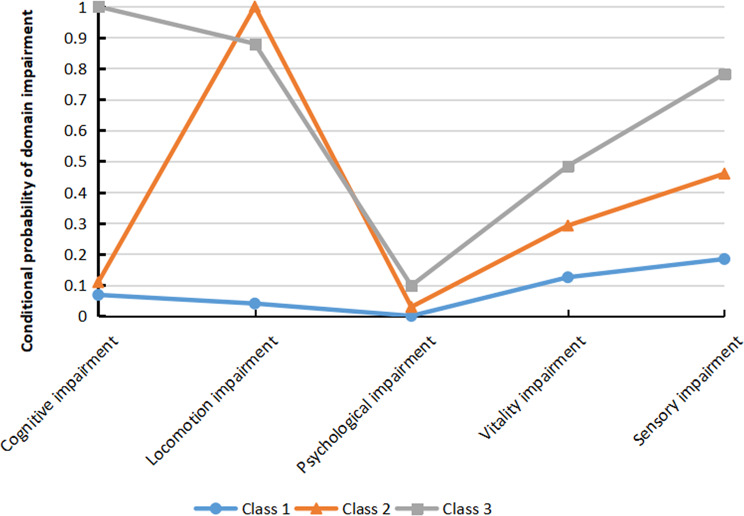
Latent profiles of IC impairment for 3-class model. Blue line indicates the latent profile of Class 1 “high IC”. Orange line indicates the latent profile of Class 2 “intermediate IC mainly locomotor impairment”. Grey line indicates the latent profile of Class 3 “low IC mainly cognitive impairment”

Three classes of IC impairment are depicted in Fig. 1. We found that older adults in class 1 had mild impairment in IC domains, so it could be defined as “high IC” (*n* = 106, 44.7%). All older adults in class 2 had impaired locomotion, which could be defined as “intermediate IC mainly locomotor impairment” (*n* = 74, 31.2%). All older adults in class 3 had cognitive impairment and severe impairment in other IC domains, thus it was defined as “low IC mainly cognitive impairment” (*n* = 57, 24.1%).

### Comparison of BADL, IADL, frailty, QOL, and falls among three classes

**Fig. 2:**
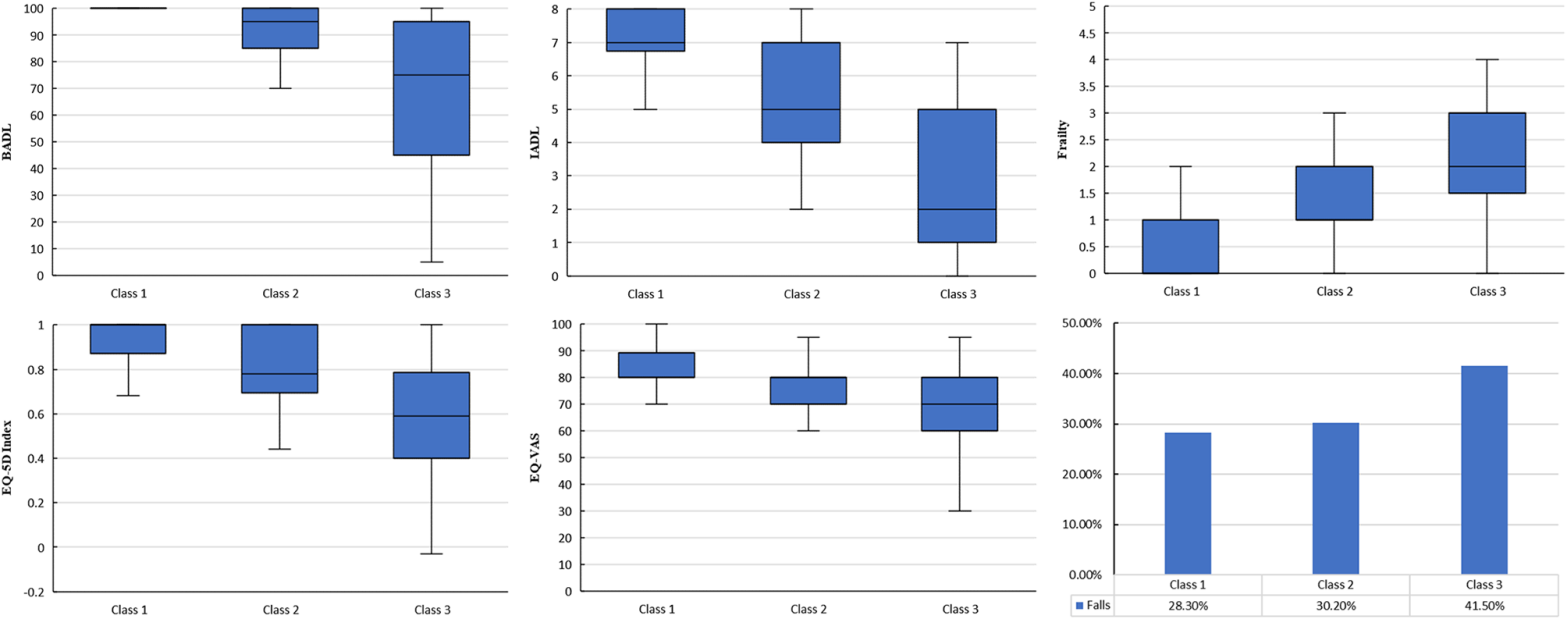
Comparison of BADL, IADL, frailty, QOL, and falls among three classes. BADL basic activities of daily living, IADL instrumental activities of daily living, EQ-5D EuroQoL-5D, VAS visual analogue scale

The characteristics of BADL, IADL, frailty, QOL (EQ-5D index and EQ-VAS), and falls among the three classes were further compared in Fig. 2 (details in Supplementary Material S1). We found that scores of BADL, IADL and EQ-5D index were highest in class 1 and lowest in class 3, while frailty scores were reversed (*P* < 0.001). Class 1 had a higher score of EQ-VAS than other classes (*P* < 0.001). The prevalence of falls was higher in the class 3 than in the class 1 (*P* = 0.002).

### Factors associated with the classes of IC impairment

The comparisons of the characteristics between the three classes of IC impairment are presented in Table 3 and statistically significant differences were found in age, living alone, nutrition, health responsibility, interpersonal relationships, physical activity, spiritual growth, stress management, polypharmacy, CCI, stroke, COPD, osteoporosis, and SSRS (*P* < 0.05). These variables were further included in multinomial logistic regression analysis as independent variables, with three classes of IC impairment as dependent variables. Table 4 presents the results of the multinomial logistic regression analysis. Older adults with advanced age (age ≥ 90 years) (*OR* = 22.046, *95%CI*:1.735-280.149), osteoporosis (*OR* = 3.377, *95%CI*:1.161–9.825), and lower scores in physical activity (*OR* = 0.842, *95%CI*:0.749–0.945), stress management (*OR* = 0.762, *95%CI*:0.585–0.993) and SSRS (*OR* = 0.897, *95%CI*:0.833–0.965) were more likely to belong to the class 2 than the class 1. Simultaneously, compared with class 1, advanced age (age ≥ 90 years) (*OR* = 104.435, *95%CI*:6.038-1806.410), stroke (*OR* = 3.877, *95%CI*:1.172–12.823) and lower scores in physical activity (OR = 0.784, *95%CI*:0.667–0.922) and SSRS (*OR* = 0.909, *95%CI*:0.828–0.998) were more likely to be class 3. In addition, compared with class 2, older adults with a lower score in nutrition (*OR* = 0.764, *95%CI*:0.615–0.950) were more likely to belong to class 3.

**Table 3:** Participants characteristics of IC impairment among three classes

Variables	Class 1 (*n* = 106)	Class 2 (*n* = 74)	Class 3 (*n* = 57)	H/χ^2^/F	*P*
Age, years, n (%)				89.485	< 0.001
60–74	47 (44.3%)	10 (13.5%)	4 (7.0%)		
75–89	58 (54.7%)	52 (70.3%)	22 (38.6%)		
≥ 90	1 (1.0%)	12 (16.2%)	31 (54.4%)		
Gender, n (%)				0.105	0.949
Male	67 (63.2%)	46 (62.2%)	37 (64.9%)		
Female	39 (36.8%)	28 (37.8%)	20 (35.1%)		
Education level, years, n (%)				7.527	0.111
< 9	20 (18.9%)	17 (23.0%)	16 (28.1%)		
9–12	24 (22.6%)	23 (31.1%)	20 (35.1%)		
> 12	62 (58.5%)	34 (45.9%)	21 (36.8%)		
Monthly personal income, n (%)				0.138	0.933
< 5000 ¥ (approximately 700 US$)	22 (20.8%)	15 (20.3%)	13 (22.8%)		
≥ 5000 ¥ (approximately 700 US$)	84 (79.2%)	59 (79.7%)	44 (77.2%)		
Living alone, n (%)				6.171	0.046
No	93 (87.7%)	58 (78.4%)	53 (93.0%)		
Yes	13 (12.3%)	16 (21.6%)	4 (7.0%)		
Current smoking, n (%)				2.487	0.288
No	92 (86.8%)	66 (89.2%)	54 (94.7%)		
Yes	14 (13.2%)	8 (10.8%)	3 (5.3%)		
Sleeping hours, M (P25, P75)	6 (4.5, 6.5)	6 (5, 7)	6 (4.5, 8)	2.818	0.244
Nutrition, M (P25, P75)	21 (19.75, 23)	21 (20, 22)	19 (17, 21)	28.938	< 0.001
Health responsibility, M (P25, P75)	23 (19.75, 27)	21 (18.75, 23.25)	18 (15, 20)	43.533	< 0.001
Interpersonal relationships, M (P25, P75)	15 (12, 18)	13 (10, 15)	10 (8, 13)	51.030	< 0.001
Physical activity, M (P25, P75)	17 (15, 20.25)	12.5 (10, 16)	9 (8, 13)	86.365	< 0.001
Spiritual growth, M (P25, P75)	10 (8, 13)	8 (6.75, 10)	6 (5, 9.5)	40.902	< 0.001
Stress management, M (P25, P75)	14 (13,15)	14 (13,14)	14 (12,14)	11.086	0.004
Polypharmacy, n (%)				8.175	0.017
No	48 (45.3%)	21 (28.4%)	15 (26.3%)		
Yes	58 (54.7%)	53 (71.6%)	42 (73.7%)		
CCI, M (P25, P75)	1 (0, 2)	1 (0, 3)	1 (1, 2)	12.099	0.002
Stroke, n (%)				12.449	0.002
No	79 (74.5%)	44 (59.5%)	27 (47.4%)		
Yes	27 (25.5%)	30 (40.5%)	30 (52.6%)		
Hypertension, n (%)				0.218	0.897
No	21 (19.8%)	16 (21.6%)	13 (22.8%)		
Yes	85 (80.2%)	58 (78.4%)	44 (77.2%)		
Diabetes, n (%)				0.210	0.900
No	65 (61.3%)	47 (63.5%)	34 (59.6%)		
Yes	41 (38.7%)	27 (36.5%)	23 (40.4%)		
Coronary heart disease, n (%)				1.759	0.415
No	75 (70.8%)	46 (62.2%)	36 (63.2%)		
Yes	31 (29.2%)	28 (37.8%)	21 (36.8%)		
COPD, n (%)				8.889	0.007
No	105 (99.1%)	66 (89.2%)	55 (96.5%)		
Yes	1 (0.9%)	8 (10.8%)	2 (3.5%)		
Osteoporosis, n (%)				8.967	0.011
No	92 (86.8%)	51 (68.9%)	42 (73.7%)		
Yes	14 (13.2%)	23 (31.1%)	15 (26.3%)		
Cancer, n (%)				2.539	0.319
No	102 (96.2%)	67 (90.5%)	53 (93%)		
Yes	4 (3.8%)	7 (9.5%)	4 (7.0%)		
SSRS, Mean ± SD	40.08 ± 6.635	33.4 ± 7.023	32.86 ± 6.242	24.039	< 0.001

CCI Charlson Comorbidity Index, COPD chronic obstructive pulmonary disease, SSRS Social support rate scale, M median, SD standard deviation

**Table 4:** Multinomial logistic regression analysis for IC impairment among three classes

Variables	Class 2 vs. class 1 ^a^		Class 3 vs. class 1 ^a^		Class 3 vs. class 2 ^a^	
	OR	95%CI	OR	95%CI	OR	95%CI
Age, years						
60–74	1.00 (reference)		1.00 (reference)		1.00 (reference)	
75–89	2.082	0.754–5.747	2.564	0.519–12.654	1.231	0.243–6.230
≥ 90	22.046	1.735-280.149	104.435	6.038-1806.410	4.737	0.797–28.170
Living alone						
No	1.00 (reference)		1.00 (reference)		1.00 (reference)	
Yes	0.418	0.126–1.382	0.236	0.047–1.187	0.563	0.141–2.249
Nutrition	1.126	0.917–1.382	0.860	0.676–1.094	0.764	0.615–0.950
Health responsibility	0.973	0.883–1.072	0.904	0.792–1.032	0.929	0.828–1.043
Interpersonal relationships	0.964	0.836–1.113	0.900	0.750–1.081	0.933	0.797–1.093
Physical activity	0.842	0.749–0.945	0.784	0.667–0.922	0.932	0.803–1.081
Spiritual growth	0.914	0.772–1.082	0.959	0.767–1.199	1.049	0.864–1.273
Stress management	0.762	0.585–0.993	0.733	0.519–1.034	0.961	0.713–1.297
Polypharmacy						
No	0.611	0.250–1.493	0.411	0.125–1.352	0.673	0.236–1.917
Yes	1.00 (reference)		1.00 (reference)		1.00 (reference)	
CCI	1.147	0.752–1.750	0.911	0.520–1.597	0.794	0.495–1.273
Stroke						
No	1.00 (reference)		1.00 (reference)		1.00 (reference)	
Yes	1.555	0.602–4.015	3.877	1.172–12.823	2.493	0.903–6.882
COPD						
No	1.00 (reference)		1.00 (reference)		1.00 (reference)	
Yes	6.502	0.532–79.440	2.011	0.094–43.068	0.309	0.039–2.441
Osteoporosis						
No	1.00 (reference)		1.00 (reference)		1.00 (reference)	
Yes	3.377	1.161–9.825	2.561	0.672–9.753	0.758	0.269–2.136
SSRS	0.897	0.833–0.965	0.909	0.828–0.998	1.014	0.939–1.095

^a^ Reference group, OR odds ratio, 95% CI 95% confidence interval, CCI Charlson Comorbidity Index, COPD chronic obstructive pulmonary disease, SSRS Social support rate scale

## Discussion

Few studies explored patterns of IC impairment, and this study identified three common IC impairment classes in hospitalized older patients using latent class analysis: 44.7% high IC (Class 1), 31.2% intermediate IC mainly locomotor impairment (Class 2) and 24.1% low IC mainly cognitive impairment (Class 3). Older adults in class 3 had the highest levels of disability and frailty, the poorest quality of life and a higher prevalence of falls. Class 2 intermediate between class 1 and 3. This is the first study to explore associated factors of IC impairment patterns, particularly multi-dimensional healthy lifestyle factors. Age, physical activity, stress management, stroke, osteoporosis, nutrition and social support were factors influencing IC impairment. Interventions should be designed according to these factors to target this three common IC impairment patterns.

The prevalence of IC impairment in this study was common (69.6%) and comparable to 73.7% as reported by a systematic review in Chinese older adults [39]. To date, some data-driven studies [40–43](using LCA/cluster analysis) have been performed. Although the number or characters of the IC impairment patterns vary between these studies because of different designs or methods, there is increasing evidence for three IC impairment patterns. We identified three patterns of IC impairment: high IC, intermediate IC mainly locomotor impairment and low IC mainly cognitive impairment, which are similar to the public-health framework proposed by WHO. Although the WHO divided older adults into three subgroups: high and stable capacity, declining capacity and significant loss of capacity, the criteria for division are unclear due to the lack of standard operation of the IC score [2]. Our study provides a new insight to understand the public-health framework for health aging. Yu et al. also identified three IC impairment patterns, including “relatively healthy”, “sharp declines in sensory domain” and “declines in locomotion, psychological, cognition and vitality domains” [41]. But Gonzalez-Bautista et al. explored the natural history of intrinsic capacity impairment over a period of 4–5 years and identified four latent statuses as follows: “high IC”, “low deterioration with impaired locomotion”, “high deterioration without cognitive impairment”, and “high deterioration with cognitive impairment” [42]. Interestingly, we found that impairment in the psychological, vitality and sensory domains tend to ‘move in block’, implying there are some correlations between these three domains. But cognition impairment and locomotion impairment tend to form independent categories, which is in line with the findings of Gonzalez-Bautista et al [42]. The locomotion impairment may be earlier and faster than other capacities and the classes characterized by cognitive impairment represent the transition to mortality [40, 42].

Our results revealed the significant associations of the classes with disability, frailty, quality of life and falls, which validate the clinical relevance of the these patterns. Underlying of intrinsic capacity lie physiological reserves, and a decline in intrinsic capacity progressively leads to frailty and ultimately to dependency [44]. Previous studies have demonstrated that older adults with lower intrinsic capacity have a higher risk of frailty, disability, poor quality of life and falls [40, 41, 45]. Therefore, it is crucial to take measures to prevent or reverse IC impairment and thus reduce adverse health outcomes.

Increased age was found to be a risk factor for more serious IC impairment. Our finding supports previous studies revealing a similar association between IC and age. Prince et al. observed that multiple IC domains impairments were more common in older age groups and all domains of IC decreased significantly with age except psychology [46]. Interestingly, we found that 7% of older adults in class 3 aged 60 to 74 years, revealing the heterogeneous trajectory of aging, which highlights that IC may be a better indicator to capture the overall health of older people than age.

Our study indicated that reduced physical activity (PA) was independently associated with more severe IC impairment. A previous study reported that less exercise (exercising for < 3 h/week) was related to IC decline [10]. In contrast, older adults engaging in moderate physical activity, vigorous physical and yoga-related activity were positively associated with high IC [9]. The benefits of PA have been proven in numerous empirical studies, including improving physical and mental capacities (e.g.,cognitive function, mobility, anxiety and depression) [47–49] and preventing and managing many chronic diseases (e.g.,hypertension, diabetes, COPD, cancer, osteoporosis, sarcopenia) [50, 51].Older adults are recommended to engage in 150 min of moderate- or 75 min of vigorous-intensity aerobic activity and two or more days of muscle-strengthening activity (e.g., strength/resistance training) per week according to the WHO’s PA guideline [52].

As for chronic diseases, older adults with osteoporosis were more likely to belong to class 2 which is primarily impaired in locomotion and sensory. Osteoporosis and fracture always appear together. Vertebral body fracture, the most common type of osteoporosis fracture, limits the mobility of the individual due to back pain when postural changes, resulting in a sedentary and inactive lifestyle that consequently accelerates disability [53]. In addition, the association between osteoporosis and hearing loss also has plausible physiological mechanisms. Osteoporosis leads to demineralization of the temporal bone, including the cochlea capsule and the conductive system, which contributes to hearing loss [54]. However, older adults with the history of stroke were more likely to belong to the low IC mainly cognitive impairment group. The relationship between stroke and cognition is evident. A Chinese cohort study revealed that incident stroke was associated with acute declines in global cognition, episodic memory, visuospatial abilities, and accelerated declines in calculation, attention, and orientation abilities [55]. Post-stroke depression, vascular cognitive impairment, post-stroke fatigue, and mobility impairments are common consequences of stroke and these conditions can overlap occurrence [56, 57], leading to multidimensional impairment of IC.

A lower stress management score was a predictor of the intermediate IC mainly locomotor impairment group. Older adults in this group are under great stress, since they are in a transition period between health and disability. In this period, they are experiencing functional decline which challenges their ability to maintain independence and social activities. Individuals without good stress management capabilities lack confidence in dealing with problems and challenges [58] and they tend to have negative self-perceptions and attitudes toward aging leading to reduced activity engagement in health behaviors and driving a downward spiral of functioning and well-being [59]. Older adults are encouraged to adopt problem-focused (using medication, healthy diet and exercise, visiting to the doctor) and emotion-focused (reframing, mindfulness) coping approaches to relieve stress [60]. Inadequate family and peer support is one of the main causes of poor stress management reported by Chinese older people [61]. Therefore, family members and friends are encouraged to provide emotional and instrumental support for older adults, such as having someone to confide in, helping with chores and providing information, advice and feedback.

A lower score in nutrition, adhering to the more unhealthy dietary pattern, was associated with belonging to low IC mainly cognitive impairment. In line with our finding, the association between dietary patterns and IC has recently been established. A research found that the “fruits and vegetables” dietary pattern and “protein-rich” dietary pattern are positively associated with IC changes, whereas following the “sugar and fat” dietary pattern is associated with decreased IC in a Japanese population-based cohort [62]. Dietary patterns might potentially affect IC through nutrition, because a better quality of diet is associated with a lower risk of malnutrition [63]. In fact, nutritional status is a candidate attribute of vitality capacity, which is a core physiological determinant of IC and the impairment of vitality may lead to a hierarchical cascade of impairments in other IC domains [64]. Aging associated physiological changes, such as teeth loss and masticatory dysfunction [65] and decreased sense of taste or smell [66], negatively impact nutritional status. Older adults with severe tooth loss and masticatory impairment tend to limit the consumption of various types of food, particularly fruits and vegetables, increase the consumption of sugary and easy-to-chew foods and decrease the intake of fiber and vitamins [67]. However, accumulating evidence suggests that high consumption of fruits, vegetables, and whole grains and low consumption of sugars and saturated fats are able to prevent cognitive decline through antioxidant and anti-inflammatory actions and other mechanisms [68]. There is a food guide pyramid for older adults, which emphasizes fluid intake, physical activity, fruits, vegetables, whole grains, animal proteins, fats, calcium and vitamins [69].

We found lower levels of social support were associated with more severe IC impairment. Social support can buffer the harmful impacts of stress on physical and mental health to maintain individuals’ well-being [70]. Previous studies revealed that social networks have broader impacts on many health outcomes, such as physical, cognitive, psychological, and overall health [71]and higher levels of social isolation were positively correlated with lower intrinsic capacity [72]. Given the considerable social relationship of older adults may change over time due to the relocation of children, relatives, or friends; and death or function limited among social networks [73]. Providing social support in time to address the unmet care needs of older adults and keep them active is warranted [74]. Social support includes formal support provided by government, institutions and communities and informal social support provided by family members, relatives, neighbors and friends [75]. Family members need to encourage older adults to develop healthy lifestyles and provide long-term support including financial support, daily care, and spiritual comfort. The community should provide visiting nursing services and voluntary social services for home-based older adults covering psychological counseling, disease monitoring, health maintenance, social intercourse, and regular visits and communication [76]. In addition to providing enough medical or pension insurance, the government should issue policies and initiatives to promote further integrated care services coordinating interdisciplinary teams and informal caregivers to provide person-centered care for older adults [77].

Lastly, from the perspective of the public-health framework [2], this study may shed light on the fact that older adults with high IC decline to intermediate IC mainly locomotor impairment stage due to aging, reduced physical activity, poor stress management, osteoporosis and lack of social support. And older adults with intermediate IC mainly locomotor impairment stage decline to low IC mainly cognitive impairment stage due to decreased nutrition intake. Although careful caution is needed and these factors should be reconfirmed through longitudinal studies, our study provides ideas for the development of intervention strategies targeting different patterns of IC impairment to reverse or delay the decline in intrinsic capacity.

### Strength and limitation

The major strength of this study is that this is the first study using a data-driven approach to explore the associated factors of IC impairment patterns. In addition, only a few studies investigated the association between lifestyle behaviors and IC. Moreover, the measurement of IC in this study was conducted in accordance with the approach recommended by the WHO. However, this study has several limitations. First, the causal relationships between IC impairment patterns and their predictors cannot be established due to the cross-sectional nature. Secondly, the sensory domain was evaluated using self-reported indicators, which may exist with reporting biases. Thirdly, considering the validity of the assessment, we excluded participants who were deaf, blind, and had severe cognitive impairment, which reduced the prevalence of impaired IC. However, this is a common situation in studies involving IC. In addition, in order to reduce nonresponsive bias, some objective information in ADL, IADL, and Frail scales was collected from primary caregivers of participants with severe hearing impairment, but it may lead to information bias although the proportion was no more than 3%. Lastly, a single hospital setting and small sample size may limit the generalizability of the findings to other populations. Therefore, further longitudinal, and multi-center studies with a larger sample size are needed.

## Conclusion

In summary, three distinct IC impairment patterns were identified: high IC, intermediate IC mainly locomotor impairment and low IC mainly cognitive impairment. Our study also identified factors associated with IC, including age, physical activity, stress management, stroke, osteoporosis, nutrition and social support, which provides insights into how to make health-promotion strategies targeted for different IC impairment patterns to delay IC decline.

### Electronic supplementary material

Below is the link to the electronic supplementary material.

Supplementary Material 1

## Data Availability

The data analyzed in this study are not publicly available but available from the corresponding author upon reasonable request.

## Abbreviations

WHO: World Health Organization
IC: Intrinsic capacity
ICOPE: Integrated Care for Older People
LCA: latent class analysis
HPLP-IIR: Health-promoting lifestyle profile-II
BADL: Basic activities of daily living
IADL: Instrumental activities of daily living
QOL: Quality of life
EQ-5D-3 L: Three-level EuroQol-5D scale
CCI: Charlson Comorbidity Index
COPD: chronic obstructive pulmonary disease
AIC: Akaike Information Criterion
BIC: Bayesian Information Criterion
SABIC: Sample-size Adjusted BIC
LMR: Lo-Mendell-Rubin
PA: Physical activity
