# Supplementary material for: Factors associated with intrinsic capacity impairment in hospitalized older adults: a latent class analysis
Source: BMC Geriatr. 2024 Jun 5;24:494. doi: 10.1186/s12877-024-05093-z (PMC11151595; doi:10.1186/s12877-024-05093-z)
Supplement: Supplementary file 1 — Supplementary Material 1 [file 12877_2024_5093_MOESM1_ESM.docx]

Supplementary Material S1. Differences in BADL, IADL, frailty, QOL, and falls among three classes

| Variables | Class 1 (n=106) | Class 2 (n=74) | Class 3 (n=57) | H/χ^2^ | P | Post-hoc |
| --- | --- | --- | --- | --- | --- | --- |
| BADL | 100 (100,100) | 95 (85,100) | 72.5 (47.5,95) | 88.922 | < 0.001 | 1>2>3 |
| IADL | 7 (6.75,8) | 5.5 (4,7) | 2 (1,5) | 106.364 | < 0.001 | 1>2>3 |
| Frailty | 0 (0,0) | 1(1,2) | 2(1,3) | 74.962 | < 0.001 | 3>2>1 |
| EQ-5D | 1 (0.87,1) | 0.78 (0.1,1) | 0.59 (0.35,0.79) | 76.604 | < 0.001 | 1>2>3 |
| EQ-VAS | 80 (80,89.25) | 80 (70,80) | 70 (60,80) | 35.325 | < 0.001 | 1>2,3 |
| Falls | 15 (28.3%) | 16 (30.2%) | 22 (41.5%) | 12.792 | 0.002 | 3>1 |

BADL basic activities of daily living, IADL instrumental activities of daily living, EQ-5D EuroQoL-5D,

VAS visual analogue scale
